# Association between diabetes mellitus, hypertension, migraine, and recurrence risk in benign paroxysmal positional vertigo: a retrospective cohort study

**DOI:** 10.3389/fneur.2026.1785013

**Published:** 2026-02-24

**Authors:** Bekir Doğan, Deniz Baklacı

**Affiliations:** 1 Department of Otorhinolaryngology, Zonguldak Atatürk State Hospital, Zonguldak, Türkiye; 2 Department of Otorhinolaryngology, Zonguldak Bülent Ecevit University Faculty of Medicine, Zonguldak, Türkiye

**Keywords:** benign paroxysmal positional vertigo, comorbidity, diabetes mellitus, hypertension, migraine, recurrence

## Abstract

**Objective:**

Benign paroxysmal positional vertigo (BPPV) is the most common cause of peripheral vertigo and can be effectively treated in most patients with canalith repositioning maneuvers. Nevertheless, recurrence remains a significant challenge in clinical practice. Although the pathophysiology of BPPV is primarily explained by mechanical mechanisms, the impact of systemic comorbidities on recurrence is not clearly defined. The aim of this study was to evaluate the association between diabetes mellitus (DM), hypertension (HT), migraine, and BPPV recurrence.

**Methods:**

In this retrospective cohort study, 300 patients aged 18 years and older who were diagnosed with BPPV were included. The diagnosis was established based on a compatible clinical history and the demonstration of characteristic positional nystagmus on Dix–Hallpike and/or supine roll tests. Clinical and demographic characteristics and canal involvement were recorded. Comorbidities were identified based on physician-documented diagnoses in electronic medical records and/or the regular use of disease-specific pharmacological treatment. Recurrence was defined as the reappearance of positional vertigo symptoms within a six-month follow-up period, accompanied by confirmatory positional nystagmus on clinical testing. Independent factors associated with recurrence were assessed using multivariable logistic regression analysis adjusted for age, sex, and canal involvement.

**Results:**

Of the included patients, 168 (56.0%) were female, and the median age was 57 years [interquartile range (IQR): 48–66]. Posterior semicircular canal involvement was the most common finding (74.3%). During the six-month follow-up period, BPPV recurrence was observed in 74 patients (24.7%). In the overall cohort, the prevalence of DM, HT, and migraine was 20.7, 28.7, and 15.3%, respectively. The prevalence of DM, HT, and migraine was significantly higher in patients with recurrence compared to those without recurrence (all *p* < 0.05). In multivariable analysis, DM [odds ratio (OR): 1.60; 95% confidence interval (CI): 1.01–2.54], HT (OR: 1.49; 95% CI: 1.01–2.32), and migraine (OR: 1.75; 95% CI: 1.05–2.92) were independently associated with BPPV recurrence.

**Conclusion:**

DM, HT, and migraine are moderately but significantly associated with BPPV recurrence. These findings suggest that BPPV should be evaluated within a broader clinical framework that considers systemic comorbidities in addition to its mechanical pathophysiology.

## 1 Introduction

Benign paroxysmal positional vertigo (BPPV) is one of the most common peripheral vestibular disorders encountered in clinical practice, characterized by brief episodes of vertigo triggered by changes in head position and accompanied by characteristic positional nystagmus (1). The fundamental pathophysiological mechanism of BPPV is attributed to the displacement of otoconia from the utricle into the semicircular canals, resulting in abnormal endolymphatic flow and inappropriate stimulation of vestibular afferents (2). Although canalith repositioning maneuvers provide rapid and effective symptom relief in most patients, the clinical course of BPPV is frequently complicated by recurrence. Systematic reviews and large cohort studies have reported recurrence rates typically ranging from 15 to 30% (3–5).

For many years, BPPV has been regarded predominantly as a purely mechanical disorder. However, accumulating evidence over recent years suggests that systemic factors and concomitant comorbidities may influence the clinical course of the disease and the risk of recurrence (6, 7). Beyond its well-established mechanical basis, BPPV has increasingly been recognized as a condition associated with systemic comorbidities, with metabolic and vascular disorders such as DM and hypertension (HT) reported to be linked to its occurrence and clinical course (8, 9). Collectively, these observations suggest that systemic conditions may contribute to a biological context that predisposes patients not only to the development of BPPV but also to its recurrence.

The association between migraine and vestibular disorders has also attracted increasing attention in recent years. Population-based and clinical studies have demonstrated a higher prevalence of vestibular symptoms and BPPV among individuals with migraine (10, 11). Migraine-related neurovascular dysregulation, trigeminovascular activation, and alterations in central sensory processing have been suggested to increase vestibular system sensitivity, thereby facilitating both the development and recurrence of BPPV (12, 13).

Recent large-scale cohort studies and systematic reviews have further indicated that comorbidities such as DM, HT, and migraine may be associated with an increased risk of BPPV recurrence (7, 14–16). Nevertheless, many of these studies have focused on a single risk factor or included heterogeneous patient populations. Consequently, investigations evaluating the combined impact of these commonly encountered comorbidities on BPPV recurrence using real-world data and adequately sized cohorts remain limited (17).

In this context, the present retrospective cohort study aimed to systematically evaluate the association between DM, HT, migraine, and BPPV recurrence in a large and well-characterized patient population.

## 2 Materials and methods

### 2.1 Study design

This study was designed as a single-center, retrospective cohort study to evaluate the association between DM, HT, migraine, and the risk of recurrence in patients diagnosed with benign paroxysmal positional vertigo (BPPV).

### 2.2 Ethical approval

The study was conducted in accordance with the principles of the Declaration of Helsinki. The study protocol was approved by the Institutional Clinical Research Ethics Committee (Approval No:2025/26). Owing to the retrospective design of the study and the use of anonymized patient data, the requirement for written informed consent was waived by the ethics committee. All data were analyzed in accordance with confidentiality principles.

### 2.3 Study population and patient selection

Electronic medical records of patients diagnosed with BPPV at our clinic between January 2020 and December 2024 were retrospectively reviewed. A total of 300 consecutive adult patients who met the inclusion criteria outlined below were included in the study.

#### 2.3.1 Inclusion criteria

Patients were eligible for inclusion if they met all of the following criteria:

1. Age ≥ 18 years;
2. A confirmed diagnosis of BPPV based on a compatible clinical history and the objective demonstration of characteristic positional nystagmus on Dix–Hallpike and/or supine roll tests;
3. Performance of a canalith repositioning maneuver appropriate for the involved semicircular canal in accordance with standard clinical practice;
4. Complete or marked improvement of symptoms following the initial treatment;
5. Availability of a minimum follow-up period of 6 months;
6. Complete clinical, demographic, and follow-up data available in the medical records.

#### 2.3.2 Exclusion criteria

Patients with any of the following characteristics were excluded from the study:

1. Clinical or neurological findings suggestive of central vertigo;
2. The presence of other concomitant vestibular disorders, such as Ménière’s disease, vestibular neuritis, or vestibular schwannoma;
3. Known central nervous system pathology, including cerebrovascular disease, demyelinating disorders, or intracranial mass lesions;
4. A history of traumatic vertigo or recent head trauma;
5. Persistent vertigo with no symptom improvement despite treatment;
6. A follow-up period of less than 6 months or insufficient clinical records.

### 2.4 Diagnosis and canal classification

The diagnosis of BPPV was established based on a detailed clinical history together with the observation of reproducible and characteristic positional nystagmus on Dix–Hallpike and/or supine roll tests. During clinical evaluation, the involved semicircular canal was classified as posterior, lateral, anterior, or multicanal. New episodes occurring in the same or a different semicircular canal during the follow-up period were considered recurrences. For statistical analyses, taking into account the sample size and the number of events, canal involvement was categorized into two groups: posterior semicircular canal and other canals.

### 2.5 Assessment of comorbidities

The presence of DM, HT, and migraine was determined based on physician-documented diagnoses recorded in the hospital electronic medical records at the time of BPPV diagnosis and/or the regular use of disease-specific pharmacological treatments. Patients without documented diagnoses or treatment information in the clinical records were considered negative for the respective comorbidity. While comorbidity status was determined based on documented diagnoses and medication use, specific standardized diagnostic criteria (e.g., ADA criteria for diabetes) were not explicitly verified for each patient due to the retrospective nature of the study. Consequently, a potential for misclassification bias regarding comorbidity ascertainment should be considered. Migraine diagnoses were based on clinical documentation by neurology or primary care specialists. However, due to the retrospective design, it was not possible to differentiate between vestibular migraine and other migraine subtypes, nor could we verify if formal International Classification of Headache Disorders (ICHD) criteria were strictly applied in all cases.

### 2.6 Outcome measure

The primary outcome of the study was BPPV recurrence, defined as the reappearance of positional vertigo symptoms within a six-month follow-up period after a phase of complete or marked symptom resolution following initial treatment, accompanied by confirmatory positional nystagmus on positional testing.

### 2.7 Statistical analysis

Statistical analyses were performed using the Statistical Package for the Social Sciences (SPSS), version 26.0 (IBM Corp., Armonk, NY, USA). The distribution of continuous variables was assessed using visual (histograms and Q–Q plots) and analytical methods. Continuous variables with a normal distribution were presented as mean ± standard deviation, whereas those with a non-normal distribution were expressed as median (interquartile range). Categorical variables were summarized as counts and percentages.

Comparisons between patients with and without recurrence were conducted using the chi-square test or Fisher’s exact test, as appropriate, for categorical variables, and the Student’s t-test or Mann–Whitney U test, depending on data distribution, for continuous variables.

To identify independent factors associated with BPPV recurrence, multivariable logistic regression analysis was performed. Variables considered clinically relevant and previously reported to be associated with BPPV recurrence in the literature (age, sex, and canal involvement) were included in the regression model to adjust for potential confounding effects. Results were reported as odds ratios (ORs) with 95% confidence intervals (CIs).

All statistical tests were two-tailed, and a *p* value < 0.05 was considered statistically significant.

## 3 Results

### 3.1 Patient characteristics

A total of 300 patients were included in the study, of whom 168 (56.0%) were female and 132 (44.0%) were male. The median age of the patients was 57 years [interquartile range (IQR): 48–66]. On clinical evaluation, posterior semicircular canal involvement was the most frequently observed finding, identified in 223 patients (74.3%). This was followed by lateral canal involvement in 62 patients (20.7%), anterior canal involvement in 5 patients (1.7%), and multicanal involvement in 10 patients (3.3%). The baseline demographic and clinical characteristics of the study population are summarized in Table 1.

**Table 1:** Baseline demographic and clinical characteristics of the study population (*n* = 300).

Variable	Value
Age, years, median (IQR)	57 (48–66)
Sex, *n* (%)	
Female	168 (56.0)
Male	132 (44.0)
Involved semicircular canal, *n* (%)	
Posterior	223 (74.3)
Lateral	62 (20.7)
Anterior	5 (1.7)
Multicanal	10 (3.3)

Data are presented as median (interquartile range) or number (%).

### 3.2 Recurrence rate

During the six-month follow-up period, confirmed BPPV recurrence was observed in 74 patients (24.7%), while 226 patients (75.3%) did not experience recurrence. In all recurrent cases, recurrence was confirmed by the reappearance of characteristic positional nystagmus on positional testing.

### 3.3 Comparison of patients with and without recurrence

Patients who experienced recurrence tended to be older than those without recurrence; however, this difference did not reach statistical significance. No significant difference was observed between the two groups in terms of sex distribution.

With respect to comorbidities, the prevalence of DM, HT, and migraine was significantly higher in patients with recurrence compared to those without recurrence. DM was present in 27.0% of patients in the recurrence group and in 18.6% of those in the non-recurrence group (*p* < 0.05). Similarly, the prevalence of HT was 38.0% in the recurrence group and 25.5% in the non-recurrence group (*p* < 0.05). Migraine prevalence was 22.5% among patients with recurrence, compared with 13.4% among those without recurrence (*p* < 0.05). Detailed comparisons between the groups are presented in Table 2.

**Table 2:** Comparison of patients with and without benign paroxysmal positional vertigo (BPPV) recurrence (*n* = 300).

Variable	No recurrence (*n* = 226)	Recurrence (*n* = 74)	*p* value
Age, years, median (IQR)	56 (47–65)	59 (50–68)	0.08
Sex, *n* (%)			
Female	124 (54.9)	44 (59.5)	0.49
Male	102 (45.1)	30 (40.5)	
Canal involvement, *n* (%)			
Posterior	170 (75.2)	53 (71.6)	0.54
Other canals	56 (24.8)	21 (28.4)	
Diabetes mellitus, *n* (%)	42 (18.6)	20 (27.0)	0.04
Hypertension, *n* (%)	58 (25.5)	28 (38.0)	0.03
Migraine, *n* (%)	30 (13.4)	17 (22.5)	0.04

Data are presented as median (interquartile range) or number (%). A *p* value < 0.05 was considered statistically significant.

Regarding canal involvement, posterior semicircular canal involvement was the most frequently observed pattern in both groups, and no significant difference in canal distribution was identified between patients with and without recurrence.

### 3.4 Multivariable logistic regression analysis

To identify independent factors associated with BPPV recurrence, multivariable logistic regression analysis was performed with adjustment for age, sex, and canal involvement. The analysis demonstrated that DM, HT, and migraine were independently associated with BPPV recurrence.

The presence of DM was associated with an approximately 1.6-fold increased risk of BPPV recurrence [odds ratio (OR): 1.60; 95% confidence interval (CI): 1.01–2.54]. HT was associated with an approximately 1.5-fold increase in recurrence risk (OR: 1.49; 95% CI: 1.01–2.32). Migraine was associated with a 1.75-fold increased risk of BPPV recurrence (OR: 1.75; 95% CI: 1.05–2.92). Detailed results of the regression analysis are presented in Table 3.

**Table 3:** Multivariable logistic regression analysis of factors associated with benign paroxysmal positional vertigo (BPPV) recurrence (*n* = 300).

Variable	OR	95% CI	*p* value
Age (years)	1.02	0.99–1.04	0.11
Female sex	1.18	0.68–2.04	0.56
Posterior canal involvement	0.92	0.51–1.66	0.78
Diabetes mellitus	1.60	1.01–2.54	0.045
Hypertension	1.49	1.01–2.32	0.048
Migraine	1.75	1.05–2.92	0.032

The model was adjusted for age, sex, and canal involvement. Results are presented as ORs with 95% CIs.

## 4 Discussion

In this retrospective cohort study, DM, HT, and migraine were found to be independently and moderately associated with recurrence of benign paroxysmal positional vertigo (BPPV). The recurrence rate observed during the six-month follow-up period (24.7%) is consistent with the recurrence rates of 20–30% reported in systematic reviews and meta-analyses (3–5, 14–16). This finding suggests that the study population and follow-up duration are comparable to those of previously published large cohorts and that the observed recurrence frequency reflects routine clinical practice.

The observed associations between DM and HT and BPPV recurrence in the present study are generally consistent with findings from previously reported cohort studies and systematic reviews. While some studies have suggested that these comorbidities are primarily associated with the development of BPPV, investigations focusing on recurrent cases have reported more pronounced associations with recurrence risk (7, 14–16). In our analysis, DM and HT were associated with an approximately 1.5-fold increase in recurrence risk, indicating that these comorbidities may play a modulatory rather than a deterministic role in recurrence. In particular, metabolic and vascular conditions such as DM and HT have been suggested to influence vestibular function and otolith homeostasis through systemic vascular and metabolic alterations rather than direct mechanical mechanisms (8, 9), may provide a biological explanation for the modest risk increase observed in our cohort.

The relationship between migraine and BPPV has been reported with more heterogeneous results in the literature. Although some studies have suggested that migraine is not an independent risk factor for BPPV, higher migraine prevalence has been reported particularly in cohorts of patients with recurrent BPPV (10, 11). The independent association between migraine and recurrence observed in our study suggests that migraine may influence the clinical course of BPPV in specific patient subgroups. Migraine-related mechanisms such as neurovascular dysregulation, trigeminovascular activation, and alterations in central sensory processing (12, 13) support a plausible pathophysiological basis for this association. In our study, migraine was analyzed as a general category. Given the significant clinical overlap between BPPV and vestibular migraine, future studies that specifically differentiate between these entities could provide a more nuanced understanding of how migraine-related mechanisms influence BPPV recurrence.

Nevertheless, the odds ratios observed in the present study, ranging between 1.5 and 1.8, indicate that BPPV recurrence cannot be explained solely by systemic comorbidities. In contrast to some reports suggesting higher risk estimates, our findings support the notion that recurrence is likely the result of a complex interplay of multiple clinical and biological factors. Age-related otoconial degeneration, structural changes in the vestibular system, and individual biological susceptibility have also been recognized as contributing factors in the clinical course of BPPV (6, 17).

Despite certain limitations related to its retrospective and single-center design, as well as the fact that potential selection bias cannot be ruled out due to the non-randomized patient recruitment process, the inclusion of consecutive patients, the use of an objective definition of recurrence, and a parsimonious multivariable analytical approach strengthen the reliability of the findings. Additionally, as this study was conducted in a tertiary setting, the patient population may reflect more complex or severe cases, which could influence both the observed recurrence rates and the prevalence of comorbidities compared to community-based cohorts. Furthermore, the reliance on electronic medical records for identifying comorbidities may have introduced some degree of misclassification bias, as we could not independently verify the diagnostic criteria used at the time of initial diagnosis for each patient. The lack of data on the duration and clinical control of comorbidities such as DM and HT. Factors such as glycemic control (e.g., HbA1c levels) and blood pressure variability were not available in our retrospective dataset. These parameters may influence the strength of the observed associations and should be addressed in future prospective studies. While many studies assess recurrence over 12–24 months, we chose a 6-month follow-up period to specifically evaluate early recurrence patterns, which are critical for immediate post-treatment counseling and intervention planning. Our findings should therefore be interpreted as reflecting early recurrence risk rather than long-term outcomes. Our study did not include several potentially relevant comorbidities and lifestyle factors such as osteoporosis, vitamin D deficiency, dyslipidemia, smoking status, or BMI due to the retrospective nature of the data collection. These variables have been reported to influence vestibular health and recurrence risk in other studies and should be considered in future research. Overall, the results of this study are in line with existing literature and demonstrate that DM, HT, and migraine exert a moderate yet clinically meaningful influence on the risk of BPPV recurrence.

## 5 Conclusion

In this retrospective cohort study, DM, HT, and migraine were shown to be independently and clinically significantly associated with recurrence of benign paroxysmal positional vertigo (BPPV). These findings suggest that, beyond being merely coexisting conditions, these comorbidities may act as systemic factors that moderately influence the clinical course of BPPV and the tendency toward recurrence.

Systematic consideration of the comorbidity profile in patients with BPPV may contribute to the identification of patient subgroups at higher risk for recurrence. Such an approach could be beneficial in clinical practice by facilitating individualized follow-up strategies and optimizing patient counseling.

However, owing to the retrospective and observational design of the study, the observed associations do not imply causality. Further prospective, multicenter, and mechanism-oriented studies are required to more comprehensively elucidate the role of these comorbidities in BPPV recurrence.

## Data Availability

The raw data supporting the conclusions of this article will be made available by the authors, without undue reservation.
